# HbtR, a Heterofunctional Homolog of the Virulence Regulator TcpP, Facilitates the Transition between Symbiotic and Planktonic Lifestyles in Vibrio fischeri

**DOI:** 10.1128/mBio.01624-20

**Published:** 2020-09-01

**Authors:** Brittany D. Bennett, Tara Essock-Burns, Edward G. Ruby

**Affiliations:** a Pacific Biosciences Research Center, University of Hawai’i—Manoa, Honolulu, Hawaii, USA; University of Connecticut

**Keywords:** *Aliivibrio*, chemotaxis, exopolysaccharide, gene regulation, luminescence, symbiosis, flagellar motility

## Abstract

The bioluminescent bacterium Vibrio fischeri forms a mutually beneficial symbiosis with the Hawaiian bobtail squid, Euprymna scolopes, in which the bacteria, housed inside a specialized light organ, produce light used by the squid in its nocturnal activities. Upon hatching, *E. scolopes* juveniles acquire V. fischeri from the seawater through a complex process that requires, among other factors, chemotaxis by the bacteria along a gradient of *N*-acetylated sugars into the crypts of the light organ, the niche in which the bacteria reside. Once inside the light organ, V. fischeri transitions into a symbiotic, sessile state in which the quorum-signaling regulator LitR induces luminescence. In this work we show that expression of *litR* and luminescence are repressed by a homolog of the Vibrio cholerae virulence factor TcpP, which we have named HbtR. Further, we demonstrate that LitR represses genes involved in motility and chemotaxis into the light organ and activates genes required for exopolysaccharide production.

## Abstract (precis)

TcpP homologs are widespread throughout the *Vibrio* genus; however, the only protein in this family described thus far is a V. cholerae virulence regulator. Here, we show that HbtR, the TcpP homolog in V. fischeri, has both a biological role and regulatory pathway completely unlike those in V. cholerae. Through its repression of the quorum-signaling regulator LitR, HbtR affects the expression of genes important for colonization of the *E. scolopes* light organ. While LitR becomes activated within the crypts and upregulates luminescence and exopolysaccharide genes and downregulates chemotaxis and motility genes, it appears that HbtR, upon expulsion of V. fischeri cells into seawater, reverses this process to aid the switch from a symbiotic to a planktonic state. The possible importance of HbtR to the survival of V. fischeri outside its animal host may have broader implications for the ways in which bacteria transition between often vastly different environmental niches.

## INTRODUCTION

The Gram-negative bacterium *Vibrio* (*Aliivibrio*) *fischeri* is a model organism for the study of biochemical processes underpinning bioluminescence, quorum sensing, and bacterial-animal symbioses. Luminescence in V. fischeri is activated by a quorum-sensing pathway that responds to high concentrations of two autoinducer molecules, *N*-(3-oxohexanoyl)-l-homoserine lactone (3-oxo-C6-HSL [1]) and *N*-octanoyl-l-homoserine lactone (C8-HSL [2]). When V. fischeri cells multiply to a certain density, 3-oxo-C6-HSL and C8-HSL accumulate to high local concentrations, initiating a signaling cascade that leads to upregulation of the regulator LuxR by the regulator LitR and consequent activation of the luciferase operon *luxICDABEG* (3–5; reviewed in reference 6).

Light production by V. fischeri is a crucial factor in the mutualistic symbiosis that it forms within the light-emitting organ of the Hawaiian bobtail squid, Euprymna scolopes (7–9). Juvenile *E. scolopes* become colonized with V. fischeri shortly after hatching into seawater containing this bacterium (10). V. fischeri cells initiate light-organ colonization through a series of steps, including chemotaxis toward *N*-acetylated sugars released by the squid (11, 12). After initial colonization of the squid light organ, the symbiosis undergoes a daily cyclic rhythm of three basic stages for the remainder of the squid’s life: during the day, V. fischeri cells grow to a high density in the crypts on carbon sources provided by the squid (13, 14); at night, the bacteria produce light that aids in camouflage for the squid (15, 16); and at dawn, ∼95% of the bacterial cells are expelled from the light organ into the seawater, where they may initiate colonization of new squid hatchlings, while the remaining ∼5% repopulate the light organ (17, 18).

Previous work indicated that, in V. fischeri cells newly expelled from the light organ, numerous genes are up- and downregulated relative to their level of expression in cells that had been planktonic for some time (19). Two of the genes upregulated in expelled cells were VF_A0473 and VF_A0474, comprising a small operon annotated as encoding homologs of the genetic regulator TcpP and its chaperone, TcpH. TcpP was first described in Vibrio cholerae as a virulence gene regulator (20, 21). During the early stages of V. cholerae infection, TcpP works synergistically with another transcriptional regulator, ToxR, to upregulate expression of the virulence regulator *toxT* and thereby activate expression of cholera toxin and the toxin-coregulated pilus in response to changing environmental conditions (21, 22). *tcpPH* expression is itself induced by AphA and AphB in response to low oxygen or acidic pH (23–25). In V. cholerae El Tor biotypes, *aphA* expression is repressed by HapR (the V. cholerae ortholog of LitR) upon initiation of quorum sensing at high cell densities (26, 27). Additionally, activation of *tcpPH* expression by AphA and AphB is inhibited by the global metabolic regulator Crp (28). This complex regulatory pathway serves to upregulate *tcpPH* and, consequently, downstream virulence factors under conditions consistent with entry into the vertebrate gut, a process reversed late in the infection cycle before the bacteria exit the host and reenter an aquatic environment (29, 30).

No published genomes of V. fischeri strains contain homologs of either *toxT* or cholera toxin genes. The genome of the model strain V. fischeri ES114 does include annotated homologs of the toxin-coregulated pilus genes *tcpFETSD* and *tcpCQBA*, but this strain is alone among the 66 currently available V. fischeri genomes to do so. The V. fischeri genes regulated by the protein product of VF_A0473 are therefore unknown, as are any upstream regulatory factors that determine under which conditions the regulator is produced and active. Here, we present evidence that VF_A0473 regulates genes governing phenotypes relevant to the switch from symbiosis to a planktonic lifestyle. We therefore rename VF_A0473 and VF_A0474, currently annotated as *tcpP* and *tcpH*, as *hbtR* (*h*a*b*itat *t*ransition *r*egulator) and *hbtC* (*h*a*b*itat *t*ransition *c*haperone), respectively. In this work, we identify the HbtR regulon and uncover aspects of *hbtRC* regulation. Further, we determine that LitR is repressed by HbtR and describe LitR-regulated phenotypes beyond luminescence that are important for light-organ colonization.

## RESULTS

### The HbtR regulon is distinct from that of TcpP.

Considering that V. fischeri and V. cholerae have significantly different lifestyles, as well as the absence in 65 of 66 sequenced V. fischeri genomes of homologs of virulence-factor genes regulated by TcpP in V. cholerae, we postulated that HbtR has a distinct function from that of TcpP. To determine the regulon of HbtR, transcriptome sequencing (RNA-seq) was performed using Δ*hbtRC* mutant strains of V. fischeri ES114 carrying either empty vector or an inducible vector with *hbtRC* under the control of the *lac* promoter. Notable among the RNA-seq results (see Table S1 in the supplemental material) is that none of the *tcp* pilus gene homologs in the V. fischeri ES114 genome were expressed at significantly different levels in Δ*hbtRC* strains carrying either empty vector or the inducible *hbtRC* vector (Table S1), ruling out the possibility that HbtR regulates the same genes in V. fischeri ES114 as TcpP does in V. cholerae.

10.1128/mBio.01624-20.4 TABLE S1 RNA-seq data file. Expression-level counts of all open reading frames (ORFs) in the V. fischeri genome. Comparison of mapped read counts for Δ*hbtRC* and *hbtRC* (induced) strains. Download Table S1, XLSX file, 0.7 MB. Copyright © 2020 Bennett et al. 2020 Bennett et al. This content is distributed under the terms of the Creative Commons Attribution 4.0 International license.

### HbtR represses *litR* expression and luminescence *in vitro*.

**FIG 1:**
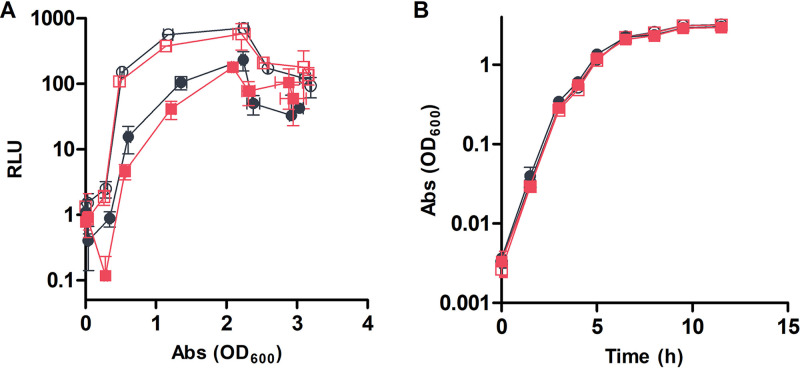
Luminescence and growth of wild-type V. fischeri and Δ*hbtRC* strains. The rates of light production (A) and growth in SWT (B) were measured for wild-type V. fischeri (circles) and Δ*hbtRC* (squares) strains carrying empty pVSV105 (open symbols) or pVSV105::*hbtRC* (closed symbols). Results represent the means from three biological replicates ±1 standard deviation. RLU, relative light units; Abs, absorbance.

Among the genes significantly downregulated in the RNA-seq results when *hbtRC* was expressed were the quorum signaling-regulated genes *litR*, *luxR*, *qsrP*, and *rpoQ* (Table 1 and Table S1). Less strongly, but statistically significantly, downregulated genes included most of the luciferase operon (Table 1 and Table S1). To determine whether repression of *lux* genes by HbtR affects bacterial light emission, luminescence and growth were monitored in cultures of wild-type V. fischeri and its Δ*hbtRC* derivative carrying either empty vector or vector constitutively expressing *hbtRC*. Deletion of *hbtRC* did not affect light production; however, overexpression of *hbtRC* in either the wild-type or Δ*hbtRC* strain led to a delay in onset and significant decrease in intensity of light production (Fig. 1A). There was no difference in growth rate between the strains (Fig. 1B), eliminating the possibility that the reduced luminescence by the *hbtRC*-overexpression strains was due to a growth defect. To determine whether deletion of *hbtRC* would lead to a change in light production by V. fischeri within the light organ, *E. scolopes* hatchlings were colonized with wild-type V. fischeri or the Δ*hbtRC* mutant. There was no difference in the luminescence of juvenile squid colonized by either V. fischeri strain at either 24 or 48 h (Fig. S1), suggesting that HbtR is not active inside the light organ.

**TABLE 1:** RNA-seq results for genes analyzed in this study^*a*^

Locus	Gene	Product description	log_2_(FC)^*b*^	*p*-adj^*c*^
Quorum-sensing genes				
VF_2177	*litR*	Quorum-sensing transcriptional regulator LitR	−1.09	1.30E−25
VF_A0919	*luxE*	Long-chain-fatty-acid ligase	−0.56	0.0002
VF_A0920	*luxB*	Luciferase beta chain	−0.39	0.007
VF_A0921	*luxA*	Luciferase alpha chain	−0.42	0.001
VF_A0922	*luxD*	Acyl transferase	−0.36	0.027
VF_A0923	*luxC*	Acyl-CoA^*d*^ reductase	−0.58	0.0005
VF_A0924	*luxI*	3-Oxo-C6-HSL autoinducer synthesis protein	−0.67	0.0005
VF_A0925	*luxR*	LuxR family transcriptional regulator	−1.29	1.78E−27
VF_A1015	*rpoQ*	Sigma-Q factor RpoQ, quorum-sensing regulated RpoS-like sigma subunit	−1.25	2.01E−14
VF_A1058	*qsrP*	LuxR family transcriptional regulator	−1.38	1.41E−05
Extracellular polysaccharide genes				
VF_0157	*wbfB*	WbfB protein	−1.36	6.75E−21
VF_0158	VF_0158	Hypothetical protein	−1.61	5.16E−23
VF_0160	*wbfD*	WbfD protein	−1.73	4.19E−14
VF_0161	VF_0161	Hypothetical protein	−1.23	4.30E−28
VF_0162	*gfcE*	Exopolysaccharide export protein	−1.98	8.73E−43
VF_0163	VF_0163	Hypothetical protein	−1.26	2.53E−17
VF_0164	*etp*	Phosphotyrosine-protein phosphatase	−2.08	1.33E−37
VF_0165	*wzc*	Protein-tyrosine kinase, chain length regulator in capsular polysaccharide biosynthesis	−1.81	2.47E−38
VF_0166	*rffG*	dTDP-glucose 4,6-dehydratase	−1.98	1.01E−45
VF_0167	*rffH*	Glucose-1-phosphate thymidylyltransferase	−1.65	1.01E−37
VF_0168	*rfbC*	dTDP-4-deoxyrhamnose-3,5-epimerase	−1.80	2.75E−38
VF_0169	*rmlB*	dTDP-glucose-4,6-dehydratase	−1.64	3.03E−31
VF_0170	*rfbX*	Polisoprenol-linked O-antigen transporter	−1.72	3.03E−24
VF_0171	VF_0171	Hypothetical protein	−1.14	1.68E−09
VF_0172	VF_0172	O-acetyltransferase	−1.64	3.26E−53
VF_0173	VF_0173	Hypothetical protein	−1.94	1.66E−33
VF_0174	VF_0174	Beta-d-GlcNAc beta-1,3-galactosyltransferase	−1.72	2.02E−36
VF_0175	VF_0175	Glycosyltransferase	−1.50	1.16E−42
VF_0176	VF_0176	3-Deoxy-8-phosphooctulonate synthase	−1.34	5.18E−30
VF_0177	VF_0177	3-Deoxy-manno-octulosonate-8-phosphatase	−1.56	1.57E−56
VF_0178	VF_0178	3-Deoxy-manno-octulosonate cytidylyltransferase	−1.60	1.91E−60
VF_0179	*kpsF*	Arabinose-5-phosphate isomerase	−1.80	3.50E−39
VF_0180	*rfe*	UDP-GlcNAc:undecaprenylphosphate GlcNAc-1-phosphate transferase	−1.78	2.39E−71
Chemotaxis and motility genes				
VF_1133	VF_1133	Methyl-accepting chemotaxis protein	1.55	4.08E−41
VF_1864	*flaC*	Flagellin	1.03	1.80E−16
VF_2042	VF_2042	Methyl-accepting chemotaxis protein	2.78	2.11E−87
VF_2079	*flaF*	Flagellin	0.93	6.75E−16
VF_A0246	VF_A0246	Methyl-accepting chemotaxis protein	1.04	8.31E−12
VF_A0389	VF_A0389	Methyl-accepting chemotaxis protein	1.20	1.34E−09

a An abbreviated subset of data in Table S1.

b Log_2_ fold change (Δ*hbtRC* + *hbtRC*/Δ*hbtRC*).

c *p*-adj, Benjamini-Hochberg adjustment of Wald test *P* value.

d CoA, coenzyme A.

10.1128/mBio.01624-20.1 FIG S1 Luminescence of wild-type V. fischeri and Δ*hbtRC* strains during symbiosis. Luminescence was measured in juvenile squid 24 h or 48 h after colonization with either wild-type V. fischeri (circles) or Δ*hbtRC* mutant (squares). Each point represents one animal. Mean values indicated; no significant differences were noted between strains at either time point. RLU, relative light units. Download FIG S1, PDF file, 0.4 MB. Copyright © 2020 Bennett et al. 2020 Bennett et al. This content is distributed under the terms of the Creative Commons Attribution 4.0 International license.

### HbtR activates and LitR represses chemosensory genes involved in *E. scolopes* light-organ colonization.

Among the genes upregulated in the *hbtRC*-expressing strain in the RNA-seq experiment were four genes annotated as encoding methyl-accepting chemotaxis proteins (MCPs): VF_1133, VF_2042, VF_A0246, and VF_A0389 (Table 1 and Table S1). To confirm that the upregulation of these MCP genes by HbtR functions through its repression of *litR* expression, reverse transcription-quantitative PCR (RT-qPCR) was performed on wild-type V. fischeri and Δ*litR* strains as well as the Δ*hbtRC* strain carrying either empty vector or vector constitutively expressing *hbtRC*. Expression of all four of these MCP genes was significantly increased in the Δ*litR* mutant compared to wild-type V. fischeri (Table 2). Expression of VF_2042 was significantly increased in the Δ*hbtRC* mutant constitutively expressing *hbtRC* compared to the Δ*hbtRC* strain carrying empty vector (Table 2), confirming the RNA-seq results indicating that HbtR affects MCP gene regulation.

**TABLE 2:** RT-qPCR results^*a*^

Gene, strain, or condition	Wild-type V. fischeri				Δ*litR* mutant			
	Δ*C_T_*^*b*^ (gene − *polA*)	SD			Δ*C_T_* (gene − *polA*)	SD	*P* value^*c*^	Fold change
Genes								
VF1133	3.01	0.03			1.12	0.07	0.001	3.70
VF2042	4.64	0.15			−1.73	0.52	0.002	86.13
VFA0246	3.87	0.06			−1.47	0.12	0.000	40.32
VFA0389	0.52	0.15			−0.81	0.18	0.002	2.52
*cheW*	−1.55	0.05			−2.53	0.24	0.019	1.99
*motA1*	0.95	0.23			−0.16	0.30	0.015	2.17
*flaC*	−1.58	0.22			−4.17	0.51	0.015	6.27
*flaF*	1.31	0.14			−1.44	0.75	0.024	7.41
*rpoN*	−0.01	0.14			0.14	0.11	0.233	0.90
*flrA*	−0.74	0.04			−0.48	0.12	0.071	0.84
*flrB*	0.85	0.13			1.38	0.63	0.293	0.74
*fliA*	−1.37	0.11			−1.81	0.27	0.119	1.38
*flgM*	−0.53	0.14			−1.35	0.31	0.053	1.80
*wbfB*	1.56	0.12			3.72	0.24	0.005	0.23
*etp*	0.38	0.09			3.42	0.08	<0.0001	0.12
*wzc*	0.35	0.20			3.46	0.17	0.0003	0.12
	**Δ*hbtRC* + pVSV105**				**Δ*hbtRC* + pVSV105::*hbtRC***			
	**Δ*C_T_* (gene − *polA*)**	**SD**			**Δ*C_T_* (gene − *polA*)**	**SD**	***P* value**	**Fold change**
Genes								
VF_2042	4.53	0.37			2.10	0.06	0.008	5.39
*etp*	−1.23	0.08			−0.26	0.08	0.001	0.51
	**Low cell density**				**High cell density**			
	**Δ*C_T_* (*hbtRC* − *polA*)**	**SD**	***P* value^***d***^**	**Fold change^***d***^**	**Δ*C_T_* (*hbtRC* − *polA*)**	**SD**	***P* value^***d***^**	**Fold change^***d***^**
Strains								
Wild-type V. fischeri	8.08	0.12			13.07	0.35	0.002	0.03
Δ*litR* mutant	8.19	0.58	0.775	0.97	13.02	0.69	0.007	0.04
Δ*crp* mutant	10.06	0.68	0.038	0.27	12.29	0.12	<0.0001	0.05
	**Low cell density**				**High cell density**			
	**Δ*C_T_* (*hbtRC* − *polA*)**	**SD**	***P* value^***e***^**	**Fold change^***e***^**	**Δ*C_T_* (*hbtRC* − *polA*)**	**SD**	***P* value^***e***^**	**Fold change^***e***^**
Conditions								
SWT alone	6.00	0.49			10.04	0.53	0.002	0.06
+ C8-HSL	5.72	0.13	0.473	1.25	10.60	0.87	0.001	0.06
+ 3-Oxo-C6-HSL	5.71	0.39	0.432	1.22	10.16	0.26	0.004	0.05

a All cultures grown in SWT.

b Δ*C_T_*, average from three biological replicates.

c *P* value, Welch’s *t* test of Δ*C_T_* values.

d *P* values and fold changes compare to wild type at low density; *P *> 0.05 for Δ*litR* and Δ*crp* mutants at high density versus wild type at high density.

e *P* value*s* and fold changes compare to wild type at low density; *P *> 0.05 for + C8-HSL and + 3-oxo-C6 at high density versus SWT alone at high density.

**FIG 2:**
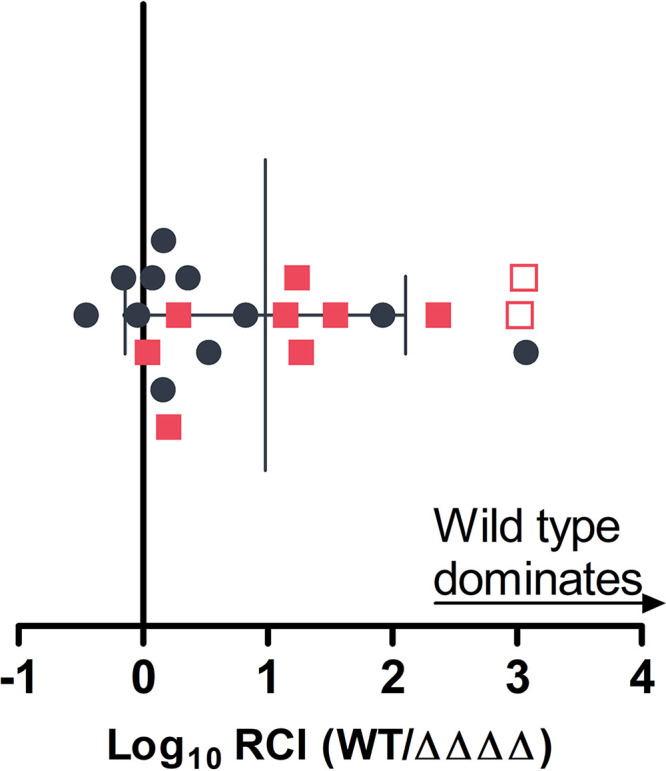
Light-organ colonization competition between wild-type V. fischeri and a chemotaxis mutant. Ratios of wild-type V. fischeri (WT) carrying a green fluorescent protein (GFP)-encoding plasmid (pVSV102) versus unlabeled ΔVF_1133 ΔVF_2042 ΔVF_A0246 ΔVF_A0389 strain (“ΔΔΔΔ”) (circles) or unlabeled wild-type V. fischeri versus the ΔΔΔΔ strain carrying pVSV102 (squares) colonizing juvenile *E. scolopes* light organs were measured after 3 h of exposure to the inoculum followed by 21 h of incubation in sterile seawater. RCI, relative competitive index of bacterial strains. Each point represents one animal. Empty symbols represent the limit of detection for light organs in which only the wild-type strain was found. Error bars represent ±1 standard deviation.

As chemotaxis is essential to colonization of the *E. scolopes* light organ by V. fischeri (12), we asked whether any of the four MCPs repressed by LitR are involved in colonization. Newly hatched *E. scolopes* juveniles were exposed to a 1:1 ratio of wild-type V. fischeri and a quadruple mutant with in-frame deletions of all four of the LitR-repressed MCP genes (ΔVF_1133 ΔVF_2042 ΔVF_A0246 ΔVF_A0389; “ΔΔΔΔ”). Regardless of which strain carried a *gfp*-labeled plasmid, the squid light organ populations tended to be dominated by wild-type V. fischeri (Fig. 2), indicating that HbtR and LitR regulate chemotaxis genes involved in initiating symbiosis.

To determine whether the colonization defect displayed by the ΔΔΔΔ strain is due to the chemosensory function of any of the four MCP genes, we sought to identify the chemoattractants recognized by these MCPs. Wild-type V. fischeri and MCP deletion mutants were spotted onto minimal medium, soft-agar plates containing 1 mM three known chemoattractants for V. fischeri (12): *N*-acetyl-d-glucosamine (GlcNAc), *N*,*N*′-diacetylchitobiose [(GlcNAc)_2_], or *N*-acetylneuraminic acid (Neu5Ac). The chemotactic zone sizes for ΔVF_1133 and ΔVF_A0246 strains were significantly smaller than for wild-type V. fischeri on each of the *N*-acetylated sugars (Table 3). Oddly, while the zone sizes of the ΔΔΔΔ strain on GlcNAc and (GlcNAc)_2_ were similar to those of the ΔVF_1133 and ΔVF_A0246 mutants, on Neu5Ac the zone size for the ΔΔΔΔ strain was comparable to that of the wild type. This is a repeatable phenotype we have yet to explain. However, a ΔVF_1133 ΔVF_A0246 mutant produced a similar zone size as the single mutants on Neu5Ac. There was no significant difference in zone sizes between the ΔΔΔΔ mutant and wild-type V. fischeri spotted onto plates containing either no chemoattractant or 1 mM glucose (Table 3), indicating these mutations do not affect motility or chemotaxis in general. Because they grew equally well on either GlcNAc or Neu5AC (Fig. S2), the differences in zone sizes of the MCP mutants and wild-type V. fischeri were unlikely to be growth related.

**TABLE 3:** Soft-agar migration zone sizes

Strain	GlcNAc			(GlcNAc)_2_			Neu5Ac		
	Zone size (mm)^*a*^	SD	*P* value^*b*^	Zone size (mm)	SD	*P* value	Zone size (mm)	SD	*P* value
Wild-type *V. fischeri*	25.7	0.6		21.7	0.6		24	1	
ΔVF_1133 mutant	18.0	0.0	<0.0001	14.0	0.0	<0.0001	18	1	0.002
ΔVF_2042 mutant	26.3	0.6	0.230	21.3	0.6	0.519	25	1.7	0.450
ΔVF_A0246 mutant	19.7	0.6	0.0002	16.3	0.6	0.0003	19.7	0.6	0.007
ΔVF_A0389 mutant	26.0	1.0	0.651	22.0	1.7	0.782	24	0	0.651
ΔVF_1133 ΔVF_A0246 mutant	ND^*c*^	ND	ND	ND	ND	ND	17.7	0.6	0.003
ΔΔΔΔ mutant	19.3	2.1	0.037	15.3	1.2	0.014	23.7	3.2	0.880
	**Glucose**			**No chemoattractant**					
	**Zone size (mm)**	**SD**	***P* value**	**Zone size (mm)**	**SD**	***P* value**			
Wild-type *V. fischeri*	18.2	0.8		17.0	0.0				
ΔΔΔΔ mutant	19.2	2.9	0.455	16.7	0.6	0.230			
	**GlcNAc**			**No chemoattractant**					
	**Zone size (mm)**	**SD**	***P* value^***d***^**	**Zone size (mm)**	**SD**	***P* value^***d***^**			
Wild-type *V. fischeri* + empty vector	10.3	1.2		6.0	1.0				
Wild-type *V. fischeri* + *hbtRC*	13.7	1.2	0.024	9.0	1.0	0.021			
Wild-type *V. fischeri* + *litR*	5.3	0.6	0.022	5.3	0.6	0.391			
Δ*litR* mutant + empty vector	18.7	0.6	0.008	17.7	1.5	0.002			
Δ*litR* mutant + *hbtRC*	20.7	0.6	0.011	18.3	1.2	0.002			
Δ*litR* mutant + *litR*	6.0	1.0	0.391	7.7	0.6	0.008			
Δ*rpoQ* mutant + empty vector	10.7	0.6	0.699	5.0	0.0	0.139			
Δ*rpoQ* mutant + *hbtRC*	15.3	1.2	0.152	9.7	1.5	0.572			
Δ*rpoQ* mutant + *litR*	4.7	0.6	0.230	5.3	0.6	1.000			

a Average from ≥3 biological replicates.

b *P* value, Welch’s *t* test compared to wild-type zones in that chemoattractant.

c ND, not done.

d *P* value for wild-type strains, compared to wild type carrying empty vector; for Δ*litR* and Δ*rpoQ* strains, compared to wild type carrying the respective vector.

10.1128/mBio.01624-20.2 FIG S2 Growth of wild-type V. fischeri and chemotaxis mutants on *N*-acetylated sugars. (A) The rates of growth in MSM supplemented with 0.05% Casamino Acids and 6.5 mM Neu5Ac were measured for wild-type V. fischeri (circles), ΔΔΔΔ (squares), and ΔVF_1133 ΔVF_A0246 (triangles) strains. (B) The rates of growth in MSM supplemented with 0.05% Casamino Acids and 6.5 mM GlcNAc were measured for wild-type V. fischeri (circles) and ΔΔΔΔ (squares) strains. Results represent means from three biological replicates ±1 standard deviation. Abs, absorbance. Download FIG S2, PDF file, 0.45 MB. Copyright © 2020 Bennett et al. 2020 Bennett et al. This content is distributed under the terms of the Creative Commons Attribution 4.0 International license.

### HbtR activates and LitR represses motility.

Two flagellin genes, *flaC* and *flaF*, were upregulated by *hbtRC* (Table 1 and Table S1), indicating the possible activation of motility by HbtR. An earlier study demonstrated that LitR represses motility in V. fischeri (31), and microarrays performed previously (S. V. Studer, A. L. Schaefer, and E. G. Ruby, unpublished data; A. L. Schaefer and E. G. Ruby, unpublished data) indicated that the C8-HSL synthase AinS and LitR downregulate the expression of a majority of the flagellin genes within the locus VF_1836–77, as well as *flaF*, *fliL2*, *motX*, *motA1B1*, and *cheW*. To confirm the repression of motility genes by LitR, RT-qPCR was employed to determine the level of expression of three flagellin or motor genes in different genomic regions in wild-type V. fischeri and Δ*litR* strains. Expression of *cheW*, *motA1*, *flaC*, and *flaF* was moderately, but statistically significantly, higher in the Δ*litR* strain than in wild-type V. fischeri (Table 2). However, we were not able to determine the mechanism by which LitR represses multiple motility gene loci, as expression levels of the known flagellin regulatory genes *rpoN*, *flrA*, *flrB*, *fliA*, and *flgM* were comparable between Δ*litR* and wild-type V. fischeri strains (Table 2).

The sigma factor-like regulator RpoQ, which is upregulated by LitR, represses motility and/or chemotaxis toward GlcNAc when overexpressed (32). Thus, we wanted to determine whether the effect LitR has on motility and *N*-acetylated sugar chemotaxis is mediated through its regulation of *rpoQ*. On soft-agar chemotaxis plates containing no chemoattractant or 1 mM GlcNAc, we observed the same pattern regardless of the presence of GlcNAc: either deletion of *litR* or overexpression of *hbtRC* led to larger migration zones than for wild-type V. fischeri, while overexpression of *litR* essentially eliminated bacterial migration (Table 3). There were no significant differences between Δ*rpoQ* and wild-type V. fischeri strains carrying the same vector (Table 3), indicating that the repression of motility by LitR is not mediated through RpoQ.

### HbtR represses and LitR activates exopolysaccharide production.

An approximately 25-kb locus constituting the genes VF_0157–80 was downregulated when *hbtRC* was expressed (Table 1 and Table S1). Previously performed microarrays indicated that most of the genes in this locus (VF_0159 and VF_0162–0180) are also upregulated by AinS and LitR (Studer et al., unpublished data; Schaefer and Ruby, unpublished). To confirm the upregulation of this locus by LitR, RT-qPCR was used to evaluate the expression of three genes, located in separate operons, in the Δ*litR* mutant and wild-type V. fischeri. Expression of VF_0157 (*wbfB*), VF_0164 (*etp*), and VF_0165 (*wzc*) was significantly higher in wild-type V. fischeri than in the Δ*litR* mutant (Table 2). Expression of *etp* was significantly lower in the Δ*hbtRC* strain constitutively expressing *hbtRC* than in the Δ*hbtRC* strain carrying empty vector (Table 2), confirming that HbtR regulation of *litR* represses gene expression in this locus.

**FIG 3:**
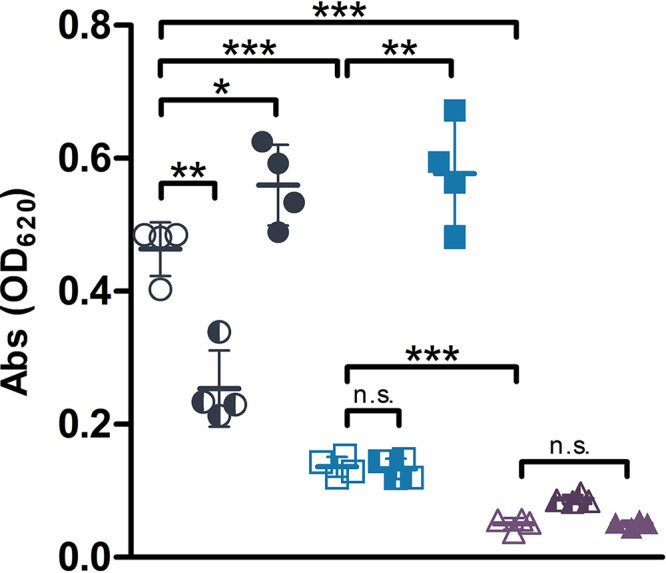
EPS production in wild-type V. fischeri, Δ*litR*, and ΔVF_0157–80 strains. EPS in supernatants of 24-h cultures grown in 0.05% Casamino Acids and 6.5 mM Neu5Ac was stained with alcian blue for wild-type V. fischeri (circles), Δ*litR* (squares), and ΔVF_0157–80 (triangles) strains carrying empty pVSV105 (open symbols), pVSV105::*hbtRC* (half-filled symbols), or pVSV105::*litR* (closed symbols). Error bars represent ±1 standard deviation. Abs, absorbance. *, *P* < 0.05; **, *P* < 0.005; ***, *P* < 0.001; n.s., not statistically significant.

Most of the genes in the locus VF_0157–80 are annotated as being involved in production of extracellular polysaccharides. However, gene annotation alone could not indicate which component(s) of the cell envelope (lipopolysaccharide O-antigen, capsule, or exopolysaccharide [EPS]) might be affected by the genes in this locus. To determine which polysaccharide type is produced by the enzymes encoded in VF_0157–80, alcian blue staining was used to detect negatively charged polysaccharides in the supernatants of liquid cultures of V. fischeri strains. Both Δ*litR* and ΔVF0157–80 strains produced less alcian blue-staining EPS than wild-type V. fischeri, with the ΔVF_0157–80 strain producing even less EPS than the Δ*litR* strain (Fig. 3). Overexpression of *hbtRC* repressed EPS production in wild-type V. fischeri, but not in the Δ*litR* or ΔVF_0157–80 strain (Fig. 3). Overexpression of *litR* in the ΔVF_0157–80 strain did not complement the reduction in EPS produced by that mutant (Fig. 3), indicating that essentially all EPS is produced by this locus.

### *hbtRC* expression is regulated by Crp but not by AphB or quorum sensing.

To determine whether *hbtRC* expression in V. fischeri is affected by quorum sensing and/or Crp, a regulator known to affect *tcpPH* expression in V. cholerae, RT-qPCR was performed on wild-type V. fischeri, Δ*litR*, and Δ*crp* strains grown to optical densities at 600 nm (OD_600_) of ∼0.3 and ∼1.0. Expression of *hbtRC* was lower at high cell density than at low cell density for all three strains, with no difference between wild-type V. fischeri and Δ*litR* strains at either higher or lower OD_600_ (Table 2). Expression of *hbtRC* in the Δ*crp* mutant was below that in wild-type V. fischeri at low OD_600_ but comparable at high OD_600_. Thus, Crp appears to activate *hbtRC* expression, the reverse of *tcpPH* regulation by Crp in V. cholerae.

Because Crp activates quorum-signaling genes in the V. fischeri genome (33, 34), we asked whether quorum signaling is responsible for the change in *hbtRC* expression at different culture densities, and whether this regulation explains the lower expression in the Δ*crp* mutant. The RT-qPCR experiment was repeated for wild-type V. fischeri grown in seawater-tryptone (SWT) with or without added autoinducers. *hbtRC* expression remained higher at lower cell density in the presence of both autoinducers (Table 2), indicating that quorum signaling is not involved in Crp- and cell density-mediated *hbtRC* regulation.

To determine whether the regulator AphAB and/or a change in pH affects *hbtRC* expression in V. fischeri, RT-qPCR was performed on RNA extracted from cultures of wild-type *V. fischeri* or an Δ*aphB* mutant grown at pH 5.5 or 8.5. There was no significant difference in *hbtRC* expression between any of the conditions (Table S2).

10.1128/mBio.01624-20.5 TABLE S2 RT-qPCR results. Download Table S2, DOCX file, 0.01 MB. Copyright © 2020 Bennett et al. 2020 Bennett et al. This content is distributed under the terms of the Creative Commons Attribution 4.0 International license.

### *tcpPH* homologs do not cross-complement between V. fischeri and V. cholerae.

Δ*tcpPH* and Δ*hbtRC* strains of V. cholerae and V. fischeri, respectively, were complemented with empty vector, vector expressing *tcpPH*, or vector expressing *hbtRC*. RT-qPCR was performed on RNA extracted from cultures of each strain to determine the level of *toxT* expression in the V. cholerae strains or *litR* expression in the V. fischeri strains. While expression of each *tcpPH* homolog complemented the respective deletion, cross-complementation had no effect on output gene expression (Table S2), illustrating the divergent activities of HbtR and TcpP.

In V. cholerae, ToxR cooperates with TcpP to activate *toxT* expression (35, 36), begging the question of whether ToxR is also involved in controlling the V. fischeri HbtR regulon. RT-qPCR performed on RNA extracted from cultures of Δ*hbtRC* and Δ*hbtRC* Δ*toxRS* mutants carrying either empty vector or vector expressing *hbtRC* showed that *litR* expression was repressed to the same degree by HbtR regardless of the presence or absence of *toxRS* (Table S2), further demonstrating that regulation by HbtR is independent of ToxR and differs substantially from the TcpP system.

### The Δ*hbtRC* mutant has no defect in colonizing juvenile *E. scolopes* light organs.

**FIG 4:**
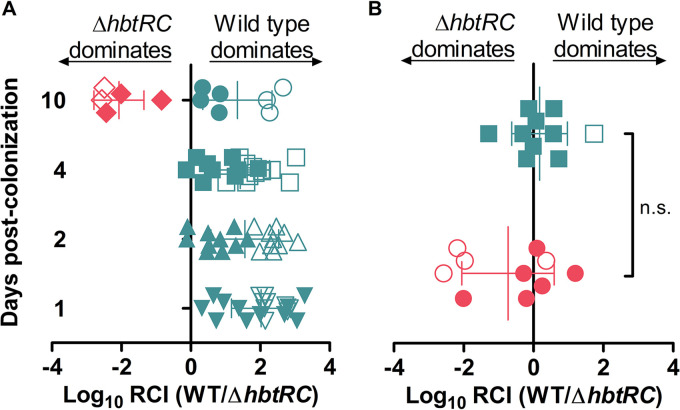
Light-organ colonization competition between wild-type V. fischeri and Δ*hbtRC* strains. (A) Ratios of wild-type V. fischeri carrying a GFP-encoding plasmid (pVSV102) versus the Δ*hbtRC* mutant carrying an RFP-encoding plasmid (pVSV208) (circles, squares, and triangles) or wild-type V. fischeri (WT) carrying pVSV208 versus the Δ*hbtRC* mutant carrying pVSV102 (diamonds) colonizing juvenile *E. scolopes* light organs were measured after 1 to 10 days. (B) Ratios of wild-type V. fischeri with chromosomal *gfp* versus Δ*hbtRC* mutant with chromosomal *rfp* (squares) or wild-type V. fischeri with chromosomal *rfp* versus Δ*hbtRC* mutant with chromosomal *gfp* (circles) colonizing juvenile *E. scolopes* light organs were measured after 1 day. RCI, relative competitive index of bacterial strains. Each point represents one animal. Empty symbols represent the limit of detection for light organs in which only the *gfp*-carrying strain was found. Error bars represent ±1 standard deviation. n.s., not statistically significant.

Previous work indicated that a Δ*hbtRC* mutant (then referred to as the Δ*tcpPH* mutant) had a light-organ colonization defect, which appeared to increase through 96 h (19). We replicated this defect when using the same conditions as the previous study (Fig. 4A); however, we observed an advantage for the Δ*hbtRC* mutant when we reversed the fluorescent marker plasmids (Fig. 4A), indicating the earlier results were due simply to experimental design. Cocolonization of juvenile *E. scolopes* with chromosomally labeled strains resulted in no significant colonization defect for either strain (Fig. 4B), demonstrating that HbtR is, in fact, not required for entry into, or life inside, the *E. scolopes* light organ. Based on this experience, we urge caution by other researchers using *rfp*-labeled pVSV208 in cocolonization experiments.

### Transcription of *hbtR* is activated as symbionts exit the light organ.

**FIG 5:**
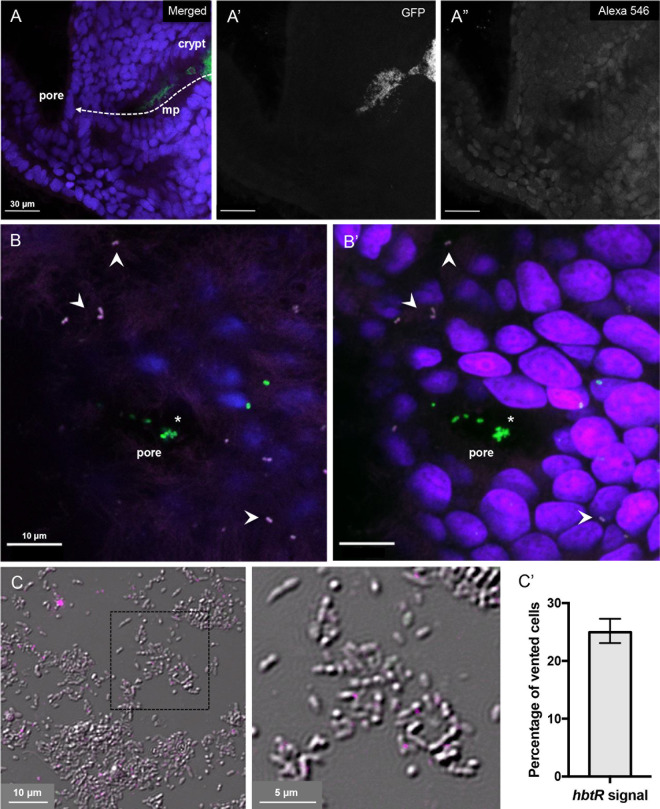
Expression of *hbtR* in V. fischeri during expulsion from *E. scolopes* light organs. Transcripts of *hbtR* (magenta, arrowheads) were detected using *in situ* HCR in V. fischeri cells (green) during expulsion from *E. scolopes* juvenile light-organ crypts. (A) V. fischeri symbionts originate in the crypt, pass through the migration path (mp), and exit through the external surface pore. (A) Merged image; (A′) GFP channel; (Aʺ) *hbtR* transcript channel (Alexa 546). (B) V. fischeri cells (green) are expelled through the external surface pore (asterisk) into seawater 1 h after a dawn light cue. (B) A single optical slice of the superficial layer above the exit point from host and V. fischeri cells outside the pore expressing *hbtR*; (B′) the corresponding merged composite stack (video in Movie S1). (C) At 1 h after expulsion into ambient seawater, vented cells express *hbtR* (magenta); enlargement is of the area within the dashed square. (C′) Percentage and 95% confidence intervals of vented cells expressing *hbtR* calculated from four samples of ventate on slides, from two experiments. Blue, *E. scolopes* nuclei (DAPI).

Considering the effects that HbtR has on chemotaxis and luminescence gene expression (Tables 1 and 2 and Table S1), and that *hbtRC* has no effect on squid luminescence (Fig. S1) or colonization (Fig. 4B), we postulated that HbtR was likely to be involved in the transition into planktonic life as V. fischeri exits the light organ. To establish at which point in the juvenile squid diel cycle V. fischeri *hbtRC* expression is activated, we performed *in situ* hybridization chain reaction (HCR) targeting *hbtR* mRNA to determine whether this gene was expressed in the bacteria before, during, or after their expulsion from the squid light organ into seawater. *hbtR* mRNA was not detected in V. fischeri cells within the crypts or during expulsion along the path out of the light organ (Fig. 5A). Only in cells that had completely exited the light-organ pores was *hbtR* expression detected (Fig. 5B and Movie S1). Within an hour of expulsion into seawater, ∼25% of expelled V. fischeri cells were expressing *hbtR* (Fig. 5C). As expected, transcripts of *litR* were detected within V. fischeri cells inside the *E. scolopes* light-organ crypts (Fig. S3); thus, it is unlikely that HCR was unable to detect *hbtR* transcripts in colonizing bacteria due to failure of the reagents to penetrate the light organ.

10.1128/mBio.01624-20.3 FIG S3 Expression of *litR* in V. fischeri when host associated within the *E. scolopes* light organ. Transcripts of *litR* (green) were detected using *in situ* HCR in V. fischeri cells colonizing *E. scolopes* crypts (C) after traversing the tissues of the migration path (MP). Blue, *E. scolopes* nuclei (DAPI); green, *litR* transcripts (Alexa 546). Bar, 15 μm. Download FIG S3, PDF file, 1.1 MB. Copyright © 2020 Bennett et al. 2020 Bennett et al. This content is distributed under the terms of the Creative Commons Attribution 4.0 International license.

10.1128/mBio.01624-20.8 MOVIE S1 Expression of *hbtR* in V. fischeri cells during their expulsion from an *E. scolopes* light organ. *hbtR* transcripts were detected using *in situ* HCR in V. fischeri cells expelled from the external surface pore of the light organ 1 h after a dawn light cue. Merged stack of images depicted in Fig. 5B′. Blue, *E. scolopes* nuclei (DAPI); green, *gfp*-labeled V. fischeri; magenta, *hbtR* transcripts (Alexa 546). Download Movie S1, AVI file, 1.5 MB. Copyright © 2020 Bennett et al. 2020 Bennett et al. This content is distributed under the terms of the Creative Commons Attribution 4.0 International license.

## DISCUSSION

**FIG 6:**
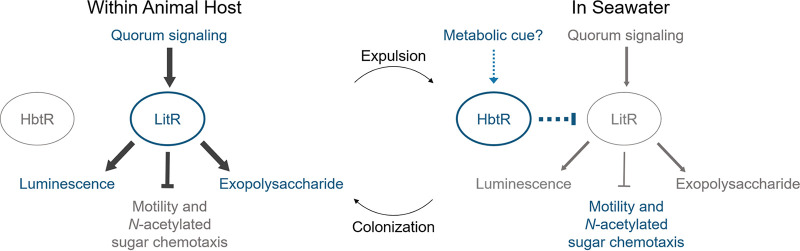
Model proposing regulation by HbtR and LitR in V. fischeri during colonization of and expulsion from the *E. scolopes* light organ. Upon colonization, LitR regulates relevant physiological phenotypes, including activation of luminescence and EPS production and repression of motility and chemotaxis. HbtR causes the repression of *litR* expression upon expulsion of V. fischeri from the light organ, reversing this process and leading to the upregulation of motility and chemotaxis and the downregulation of luminescence and EPS production. Gray text, downregulated genes and phenotypes; blue text, upregulated genes and phenotypes.

While much research has gone into understanding the mechanisms necessary for colonization of the *E. scolopes* light organ by V. fischeri (reviewed most recently in reference 37), there has been little investigation into how the ∼95% of symbiotic bacteria expelled from the light organ at dawn each day transition back into life in seawater. In this work we present HbtR, a heterofunctional homolog of the V. cholerae virulence regulator TcpP that represses the quorum-signaling regulator LitR. While it is unclear at this time whether HbtR represses *litR* expression directly or through some intermediate regulator(s), the RNA-seq data indicate HbtR does not act through a step in the quorum-signaling pathway upstream of LitR (see Table S1 in the supplemental material). Through the discovery of additional functions regulated by LitR, namely, chemotaxis, motility, and EPS production, we have begun to build a picture in which LitR aids the transition by V. fischeri into a colonization lifestyle and, upon expulsion from the light organ, HbtR reverses that process to help transition back into planktonic life (Fig. 6). This is a markedly different role for HbtR from that of TcpP.

The regulatory mechanisms controlling *hbtRC* expression, as elucidated thus far, are similar to the *tcpPH* regulatory pathway in V. cholerae only in that both operons are regulated by the global regulator Crp, albeit in the opposite manner. Notably, the *hapR* homolog in V. fischeri, *litR*, is itself repressed by HbtR (Table 1 and Tables S1 and S2), a reversal of roles from those in V. cholerae. Considering the distinct regulatory pathways for HbtR and TcpP, as well as the low percent identity (26.8%) of the two protein sequences, it appears, evolutionarily, that either progenitors of *tcpPH* and *hbtRC* were acquired independently after separation of the two species’ ancestors, or the parent operon evolved in dramatically different fashions as the two species evolved to inhabit disparate environmental niches. Determining which of these histories is the more likely is beyond the scope of this study, but in either case it is clear the two homologs have been recruited into specialized roles befitting the two species’ distinct lifestyles, i.e., pathogenesis versus beneficial symbiosis within animal hosts.

The data presented here suggest HbtR acts as a first-wave responder as V. fischeri bacteria exit the light organ and reenter the seawater (Fig. 5), a significant change in environmental conditions and an imperative for survival. This role could explain some of the discrepancies observed between the RNA-seq results in the present study and those in the work of Thompson et al. (19). For example, the Thompson et al. data indicate that in addition to *hbtR* and *hbtC*, the luminescence genes *luxR*, *luxI*, and *luxCDABEG* were also highly upregulated in cells recently expelled from the light organs compared to planktonic cells, while *litR*, which activates the *lux* genes, was not differentially regulated between the two cell populations. Meanwhile, the results of this study indicate that HbtR represses LitR and consequently causes the downregulation of *luxR* and other downstream quorum-signaling genes (Table 1 and Tables S1 and S2). Similarly, the gene most highly upregulated by HbtR in the RNA-seq data presented here was VF_2042 (Table S1), one of the four MCP genes repressed by LitR (Table 2); in the earlier study, VF_2042 was downregulated in cells released from the light organs (19). These results are consistent with a model in which HbtR becomes active once V. fischeri exits the light organ, with some of the earliest effects seen in its repression of *litR*. In the earlier study, it may have been that in the ∼25 min between initiation of light-organ expulsion and harvesting the V. fischeri RNA, HbtR had become activated (Fig. 5) and was already repressing *litR* expression, but the downstream effects (e.g., on VF_2042 and *lux* gene expression) were not yet apparent.

Previous studies have also demonstrated a repression of several flagellin genes by AinS and increased motility in a *litR* mutant (31). Here, we show that LitR represses numerous flagellin, motor, and chemotaxis genes, including at least two MCP genes involved in chemotaxis toward (GlcNAc)_2_, an *N*-acetylated sugar reported to be an important chemoattractant during light-organ colonization (12). Previous work indicated that a *litR* mutant colonized *E. scolopes* to a lower level than wild-type V. fischeri (31), which the authors suggested was due to an initiation delay due to hypermotility. However, in the study in which LitR was first described (4), a *litR* mutant had an advantage in initiating light-organ colonization over wild-type V. fischeri. Based on work presented here, including a demonstration that MCP genes repressed by LitR are involved in light-organ colonization (Fig. 2), we posit that the *litR-*mutant colonization benefit (4) may have resulted from the increase in both motility and chemotaxis into the light organ by that mutant. We hypothesize that the lower level of colonization observed for the *litR* mutant in single-strain experiments (31) may have been due to dysregulation of a separate symbiotic process, perhaps extracellular polysaccharide production.

LitR appears to activate, and HbtR to thus repress, a large locus of genes (VF_0157–80) involved in extracellular polysaccharide production (Table 1 and Tables S1 and S2). Fidopiastis et al. (4) noted that *litR*-minus colonies are less opaque than wild-type V. fischeri and suggested this phenotype may be due to a difference in the extracellular polysaccharides present in each strain. The nature of polysaccharide affected by VF_0157–80 is not fully established, yet it seems most likely to be an EPS, or “slime layer,” due to the ease with which it separates from the bacterial cell during centrifugation (Fig. 3). While lipopolysaccharide and the *syp* polysaccharide have been implicated in the initiation of host colonization by V. fischeri (38–40), little has been determined regarding the possible involvement of extracellular polysaccharides in colonization persistence. V. fischeri-colonized crypts, despite having no goblet cells (S. V. Nyholm, personal communication), have been shown to stain alcian blue positive (41), indicating that the presumptive EPS made by VF_0157–80 may be a relevant factor in *E. scolopes*-V. fischeri symbiosis. This aspect of the symbiotic relationship could have broader implications for host-microbe interactions in general.

## MATERIALS AND METHODS

### Bacterial strains and growth conditions.

Bacterial strains and plasmids used in this study are summarized in Table S3 in the supplemental material. E. coli and V. cholerae were grown in lysogeny broth (LB) (19, 42) at 37°C; V. fischeri was grown in LB-salt (LBS) (43) at 28°C. Overnight cultures were inoculated with single colonies from freshly streaked −80°C stocks. Liquid cultures were shaken at 225 rpm. Unless otherwise noted, 15 g of agar was added per liter for solid media. Antibiotics were added to overnight cultures, where applicable, at the following concentrations: kanamycin (Km), 50 μg/ml; chloramphenicol (Cm), 5 μg/ml (V. fischeri) or 25 μg/ml (E. coli). Experimental cultures were grown in either seawater-tryptone (SWT) medium (44) or minimal-salts medium {MSM; per liter: 8.8 g NaCl, 6.2 g MgSO_4_·7H_2_O, 0.74 g CaCl_2_·2H_2_O, 0.0204 g H_2_NaPO_4_, 0.37 g KCl, 8.66 g PIPES [piperazine-*N*,*N*′-bis(2-ethanesulfonic acid)] disodium salt, 0.65 mg FeSO_4_·7H_2_O; pH 7.5} supplemented with 5 mg/liter Casamino Acids and a carbon source as indicated. The results of all experiments are reported as the means from three (or more, as indicated) biological replicates ± 1 standard deviation (SD). Statistical analysis was performed using Welch’s *t* test.

10.1128/mBio.01624-20.6 TABLE S3 Bacterial strains and plasmids used in this work. Download Table S3, DOCX file, 0.03 MB. Copyright © 2020 Bennett et al. 2020 Bennett et al. This content is distributed under the terms of the Creative Commons Attribution 4.0 International license.

### Plasmid and mutant construction.

Primers used to construct the deletion and expression vectors in this study are listed in Table S4. In-frame deletion of genes from the V. fischeri genome was performed as previously described (45, 46). Counterselection to remove the target gene and pSMV3 was performed on LB-sucrose (per liter: 2.5 g NaCl, 10 g Bacto tryptone, 5 g yeast extract, 100 g sucrose) for 2 days at room temperature. Expression vectors were constructed by amplifying and ligating the target gene into the multiple cloning site of pVSV105 (47). Inducible expression of *hbtRC* was achieved by ligating *lacI*^q^ from pAKD601 (58) to *hbtRC* cloned from the V. fischeri genome and inserting the fusion into the multiple cloning site of pVSV105. Overexpression of *hbtRC* brought the level of expression closer to that found upon expulsion from juvenile squid (19), which was higher than we measured for wild-type V. fischeri under culture conditions. Genomic insertion of *gfp* or *rfp* under the control of the *lac* promoter at the *att*Tn*7* site was performed using a mini-Tn*7* vector as described previously (48). A previously constructed Δ*hbtRC* mutant (19) was used to recapitulate conditions for follow-up on previous experiments (RNA-seq and squid cocolonization); a newly derived Δ*hbtRC* strain was used for all other experiments so it would have the same parent wild-type V. fischeri stock as other mutants created in this study.

10.1128/mBio.01624-20.7 TABLE S4 Primers and probes used in this work. Download Table S4, DOCX file, 0.02 MB. Copyright © 2020 Bennett et al. 2020 Bennett et al. This content is distributed under the terms of the Creative Commons Attribution 4.0 International license.

### Generation and analysis of RNA-seq libraries.

RNA extraction and RNA-seq were performed as previously described (19). Briefly, RNA was stabilized with RNAprotect bacterial reagent (Qiagen) and extracted with an RNeasy minikit (Qiagen) from 500 μl of cultures grown to mid-log phase in SWT with 1.75 mM isopropyl-β-d-thiogalactopyranoside (IPTG). RNA was extracted from biological triplicates for each strain. Contaminating DNA was degraded by treatment with Turbo DNase (Invitrogen). Ribosomal reduction, strand-specific library preparation, and paired-end 50-bp sequence analysis on an Illumina HiSeq 2500 on high-output mode were performed at the University of Minnesota Genomics Center. Sequences were processed on the open-source Galaxy server (usegalaxy.org) (49) using the following workflow (default settings used unless otherwise indicated): reads were trimmed with Trimmomatic (maximum mismatch:1, LEADING:3, TRAILING:3, SLIDINGWINDOW:4,20, MINLEN:35), mapped to the V. fischeri chromosomes and plasmid (accession numbers NC_006840.2, NC_006841.2, and NC_006842.1) using Bowtie2 (-un-con, –sensitive), and counted with featureCounts (-p disabled, -t = gene, -g = locus_tag, -Q = 10, -s); differential expression was analyzed with DESeq2 (outliers filtering and independent filtering turned on). Between 11.7 million and 12.7 million paired reads were mapped to the V. fischeri genome per biological replicate.

### Reverse transcription and quantitative PCR.

RNA from bacterial cultures was extracted as described above and treated with Turbo DNase (Invitrogen). cDNA was synthesized from DNase-treated RNA using Smart Moloney murine leukemia virus (MMLV) reverse transcriptase (Clontech) and random hexamer primer (Thermo Scientific). Gene expression was measured by qPCR performed with LightCycler 480 SYBR green I Master Mix (Roche) under the following conditions: 95°C for 5 min; 45 cycles of 95°C for 10 s, 60°C for 20 s, and 72°C for 20 s; and melting curve acquisition from 65°C to 97°C. qPCR primer pairs were designed for these conditions and confirmed to have efficiencies between 90 and 110%. Cycle thresholds (*C_T_*) for each sample were normalized to those of the reference gene *polA* (Δ*C_T_* = target gene − *polA*; *polA* expression primers provided courtesy of Silvia Moriano-Gutierrez). ΔΔ*C_T_* values were calculated by subtracting the average Δ*C_T_* for the parent strain, at higher pH or lower OD_600_ when appropriate; fold changes were calculated as 2^(−ΔΔ^*^CT^*^)^.

### Growth and luminescence curves.

Overnight LBS cultures of each strain were pelleted, washed once, and resuspended in 1 ml of the intended growth medium. Luminescence growth curves were performed in 20 ml SWT in flasks shaking at 225 rpm; periodic 1-ml and 300-μl aliquots were taken for luminescence and OD_600_ readings on a luminometer (Turner Designs) and Tecan Genios plate reader, respectively. Growth curves were performed in 1.2 ml growth medium and monitored in the Tecan Genios plate reader with continuous shaking at high speed.

### Squid colonization assays.

Juvenile squid colonization experiments were performed as described previously (50), with juvenile squid exposed to V. fischeri strains at 1,000 to 6,000 CFU/ml either for 3 h or overnight, as indicated, before being placed in fresh sterile seawater until euthanizing by freezing. Colonization competition experiments were performed by exposing juvenile squid to a 1:1 inoculum of each strain.

### *In situ* HCR.

Hybridization chain reaction (HCR) (51) was performed on squid colonized with wild-type V. fischeri carrying a *gfp*-labeled plasmid and on V. fischeri cells 1 h after being expelled from squid light organs as previously described (52–54). Briefly, *E. scolopes* juveniles and expelled V. fischeri cells were fixed in 4% paraformaldehyde in marine phosphate-buffered saline either before or after a light cue-stimulated bacterial expulsion. HCR was performed on dissected light organs (52) and expelled V. fischeri cells affixed to gelatin-coated slides (adapted from reference 53) using HCR version 3.0 chemistry (54). Ten probes targeting *hbtR* mRNA and 11 probes targeting *litR* mRNA (Table 3) were amplified with Alexa Fluor 546-labeled hairpins (Molecular Instruments). Light organs were then counterstained overnight with a 1:750 dilution of 4′,6-diamidino-2-phenylindole (DAPI; Thermo Fisher Scientific) in 5× SSC-Tween (1× SSC is 0.15 M NaCl plus 0.015 M sodium citrate) before being mounted on slides with Vectashield (Vector Laboratories) and overlaid with a coverslip (no. 1.5, Fisherbrand; Fisher Scientific). Imaging was done on an upright Zeiss LSM 710 laser-scanning confocal microscope at the University of Hawai’i Kewalo Marine Laboratory; images were analyzed using FIJI (ImageJ) (55).

### Motility and chemotaxis assays.

Soft-agar motility assays were performed as previously described (56). Briefly, the equivalent of 10 μl of a bacterial culture grown to an OD_600_ of 0.5 in SWT was spotted onto minimal-medium plates containing 0.25% agar and, when appropriate, 1 mM chemoattractant. Migration zones were measured after 18 to 24 h of static incubation at 28°C.

### Alcian blue detection of extracellular polysaccharides.

EPS was detected by staining with alcian blue essentially as previously described (57). Single colonies from freshly streaked −80°C stocks were used to inoculate MSM supplemented with 6.5 mM Neu5Ac and 0.05% (wt/vol) Casamino Acids and grown with shaking for 18 to 22 h. EPS was separated from cells by centrifugation of the equivalent of 2.5 ml at OD_600_ = 1.0 for 15 min at 12,000 × *g* and 4°C. Two hundred fifty microliters of this supernatant was mixed with 1 ml alcian blue solution (per liter: 0.5 g alcian blue, 30 ml glacial acetic acid, pH 2.5), rocked for 1 h at room temperature, and then centrifuged for 10 min at 10,000 rpm and 4°C. Pellets were resuspended in 1 ml 100% ethanol and then centrifuged for 10 min at 10,000 × *g* and 4°C. Pellets were solubilized in 500 μl SDS (per liter: 100 g sodium dodecyl sulfate, 50 mM sodium acetate, pH 5.8), and the absorbance was read at OD_620_.

### Data availability.

Raw and processed read files have been deposited into the NCBI Gene Expression Omnibus server (GSE151621).
