# Supplementary material for: HbtR, a Heterofunctional Homolog of the Virulence Regulator TcpP, Facilitates the Transition between Symbiotic and Planktonic Lifestyles in Vibrio fischeri
Source: mBio. 2020 Sep 1;11(5):e01624-20. doi: 10.1128/mBio.01624-20 (PMC7468203; doi:10.1128/mBio.01624-20)
Supplement: FIG S1 [file mBio.01624-20-sf001.pdf]

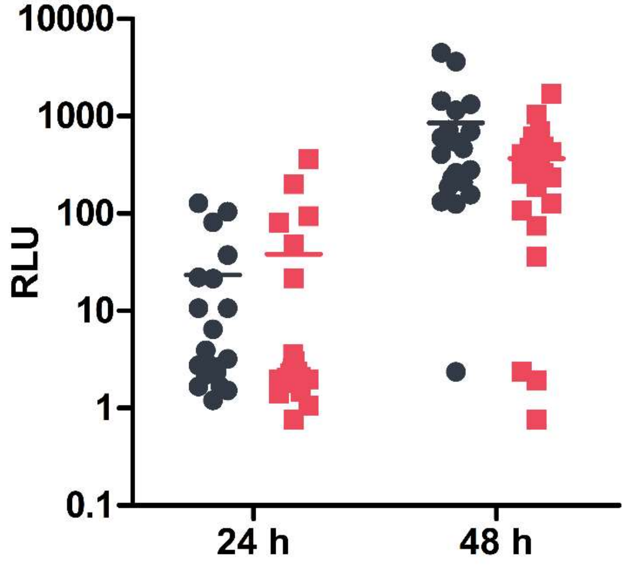

FIG S1 Luminescence of wild-type *V. fischeri* and the Δ*hbtRC* mutant during symbiosis. Luminescence was measured in juvenile squid 24 h or 48 h after colonization with either wild-type *V. fischeri* (circles) or the Δ*hbtRC* mutant (squares). Each point represents one animal. Mean values indicated; no significant differences were noted between strains at either time-point. RLU, relative light units.
