# Supplementary material for: HbtR, a Heterofunctional Homolog of the Virulence Regulator TcpP, Facilitates the Transition between Symbiotic and Planktonic Lifestyles in Vibrio fischeri
Source: mBio. 2020 Sep 1;11(5):e01624-20. doi: 10.1128/mBio.01624-20 (PMC7468203; doi:10.1128/mBio.01624-20)
Supplement: FIG S2 [file mBio.01624-20-sf002.pdf]

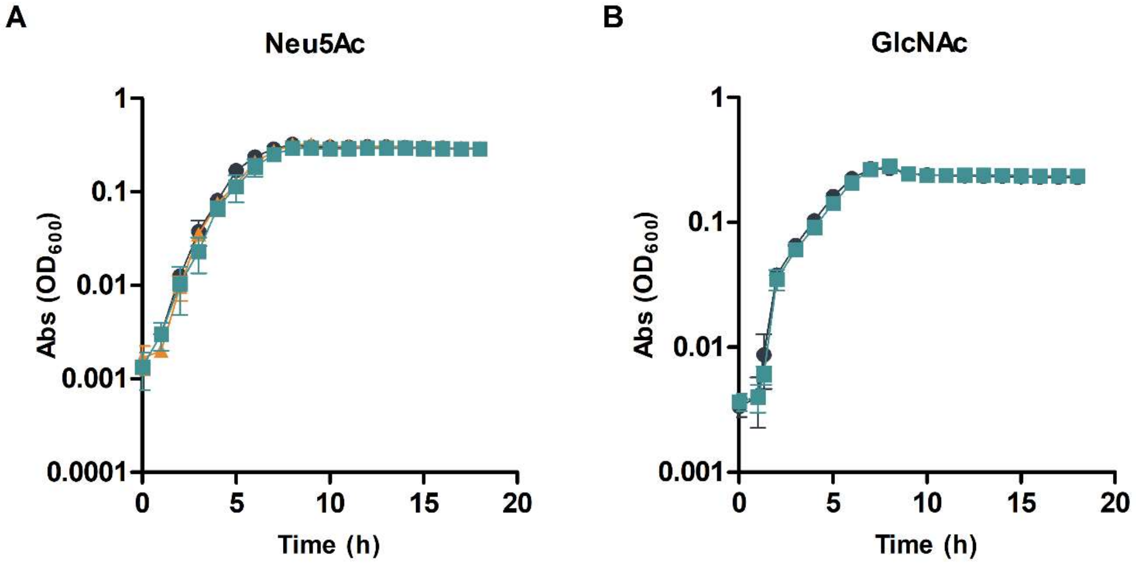

FIG S2 Growth of wild-type *V. fischeri* and chemotaxis mutants on *N*-acetylated sugars. (A) The rates of growth in MSM supplemented with 0.05% casamino acids and 6.5 mM Neu5Ac were measured for wild-type *V. fischeri* (circles), the ΔΔΔΔ mutant (squares), and the ΔVF_1133 ΔVF_A0246 mutant (triangles). (B) The rates of growth in MSM supplemented with 0.05% casamino acids and 6.5 mM GlcNAc were measured for wild-type *V. fischeri* (circles) and the ΔΔΔΔ mutant (squares). Results represent means of three biological replicates ± one standard deviation. Abs, absorbance.
