# Supplementary material for: HbtR, a Heterofunctional Homolog of the Virulence Regulator TcpP, Facilitates the Transition between Symbiotic and Planktonic Lifestyles in Vibrio fischeri
Source: mBio. 2020 Sep 1;11(5):e01624-20. doi: 10.1128/mBio.01624-20 (PMC7468203; doi:10.1128/mBio.01624-20)
Supplement: TABLE S2 [file mBio.01624-20-st002.docx]

**Table S2. RT-qPCR results**

|  | Wild-type *V. fischeri* | | | | | Δ*aphB* | | | | | |  |
| --- | --- | --- | --- | --- | --- | --- | --- | --- | --- | --- | --- | --- |
| **pH^a^** | **Δ*C_T_*  (*hbtRC* − *polA*)** | **SD** | ***P* value****^b^** | **Fold change^b^** | **Δ*C_T_*  (*hbtRC* − *polA*)** | | **SD** | ***P* value^b^** | | **Fold change^b^** | | |
| 8.5 | 10.94 | 0.37 |  |  | 10.42 | | 0.33 | 0.167 | 1.46 | |  |  |
| 5.5 | 10.55 | 0.14 | 0.227 | 1.32 | 10.34 | | 0.34 | 0.132 | 1.54 | |  |  |

| **Strain^c^** | **Δ*C_T_* (*litR*** **− *polA*)** | | | | | **SD** | | | | ***P* value** | | | | | | | **Fold change** | | | | |  |  |  |  |  |
| --- | --- | --- | --- | --- | --- | --- | --- | --- | --- | --- | --- | --- | --- | --- | --- | --- | --- | --- | --- | --- | --- | --- | --- | --- | --- | --- |
| Δ*hbtRC* strain  + empty vector | -4.38 | | | | | 0.19 | | | |  | | | | | | |  | | | | |  |  |  |  |  |
| Δ*hbtRC* strain + *hbtRC* | -3.46 | | | | | 0.42 | | | | 0.032 | | | | | | | 0.54 | | | | |  |  |  |  |  |
| Δ*hbtRC* strain + *tcpPH* | -4.65 | | | | | 0.05 | | | | 0.131 | | | | | | | 1.20 | | | | |  |  |  |  |  |
|  | | |  | | | |  | | | | |  | | | | | |  | | | | | |  |  |  |
|  | | |  | | |  | | | | | | | |  | | | | | |  | | | | | |  |
| **Strain^d^** | | | **Δ*C_T_* (*toxT* − *polA*)** | | **SD** | | | | | | ***P* value** | | | | | **Fold change** | | | | | | |  |  |  |  |
| Δ*tcpPH* strain  + empty vector | | | 4.90 | | 0.40 | | | | | |  | | | | |  | | | | | | |  |  |  |  |
| Δ*tcpPH* strain + *hbtRC* | | | 4.61 | | 0.52 | | | | | | 0.498 | | | | | 1.27 | | | | | | |  |  |  |  |
| Δ*tcpPH* strain + *tcpPH* | | | -0.06 | | 0.28 | | | | | | 0.0004 | | | | | 31.65 | | | | | | |  |  |  |  |
|  | |  | | | | | | |  | | | | | |  | | | | | |  | | | | | |
|  | | | |  | | | |  | | | | |  | | | | | |  | | | | | |  |  |
|  | | | |  | | | |  | | | | |  | | | | | |  | | | | | |  |  |
| **Strain^c^** | **Δ*C_T_* (*litR* − *polA*)** | | | | | **SD** | | | | ***P* value** | | | | | | | **Fold change** | | | | |  |  |  |  |  |
| Δ*hbtRC* strain  + empty vector | -2.19 | | | | | 0.17 | | | |  | | | | | | |  | | | | |  |  |  |  |  |
| Δ*hbtRC* strain + *hbtRC* | -0.29 | | | | | 0.32 | | | | 0.003 | | | | | | | 0.27 | | | | |  |  |  |  |  |
| Δ*hbtRC* Δ*toxRS* strain + empty vector | -1.57 | | | | | 0.96 | | | | 0.188 | | | | | | | 1.19 | | | | |  |  |  |  |  |
| Δ*hbtRC* Δ*toxRS* strain  + *hbtRC* | -0.72 | | | | | 0.26 | | | | 0.004 | | | | | | | 0.37 | | | | |  |  |  |  |  |

^a^ Cultures grown in MSM buffered to indicated pH and supplemented with 7.5 mM GlcNAc and 0.05% casamino acids

^b^ *P* values and fold changes compare to wild type at pH 8.5; *P* > 0.05 for Δ*aphB* vs. wild type at pH 5.5

^c^ Cultures grown in SWT
^d^ Cultures grown in LB
