# Supplementary material for: HbtR, a Heterofunctional Homolog of the Virulence Regulator TcpP, Facilitates the Transition between Symbiotic and Planktonic Lifestyles in Vibrio fischeri
Source: mBio. 2020 Sep 1;11(5):e01624-20. doi: 10.1128/mBio.01624-20 (PMC7468203; doi:10.1128/mBio.01624-20)
Supplement: FIG S3 [file mBio.01624-20-sf003.pdf]

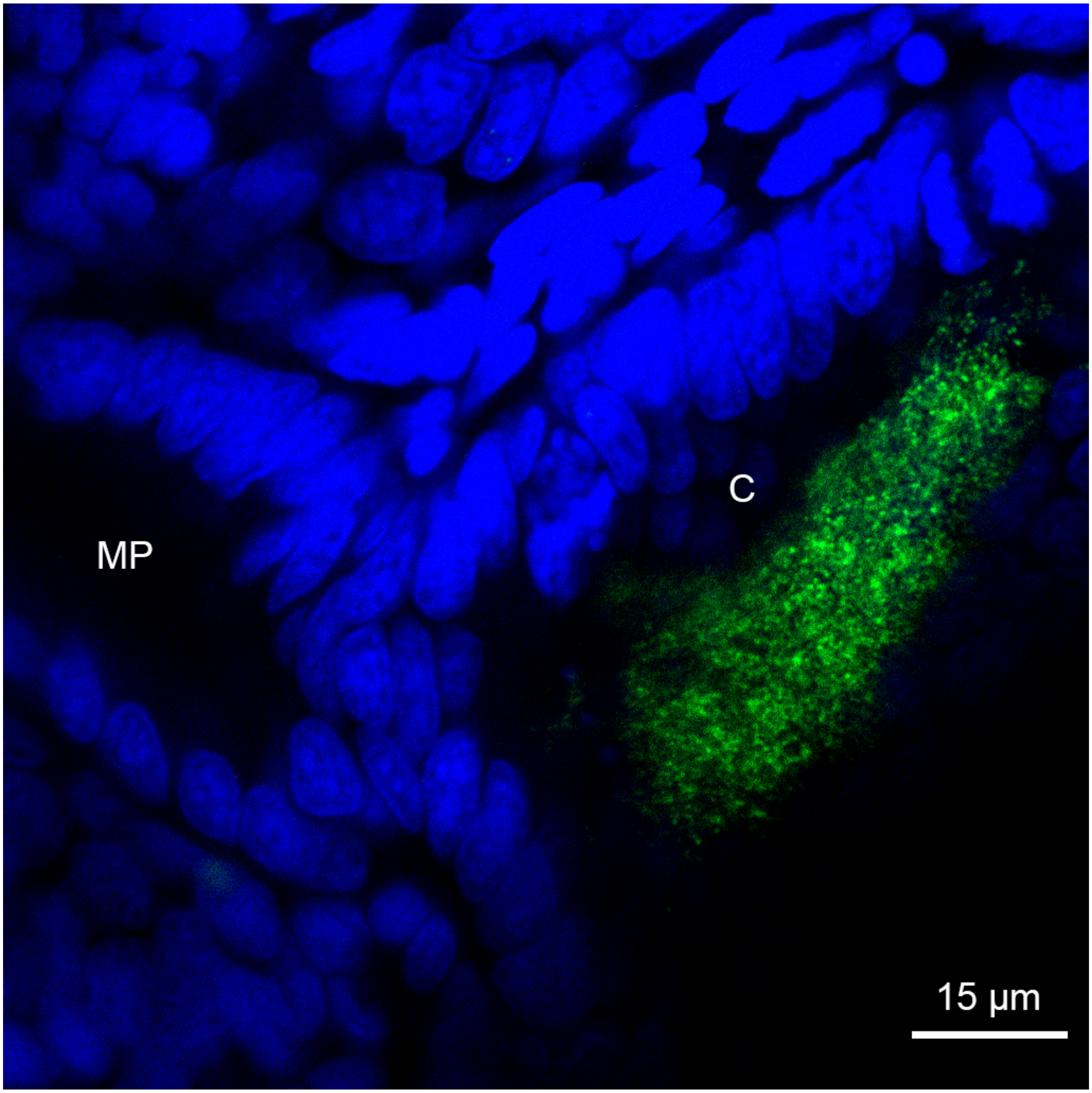

FIG S3 Expression of *litR* in *V. fischeri* when host-associated within the *E. scolopes* light organ. Transcripts of *litR* (green) were detected using *in situ* HCR in *V. fischeri* cells colonizing *E. scolopes* crypts (C) after traversing the tissues of the migration path (MP). Blue, *E. scolopes* nuclei (DAPI); green, *litR* transcripts (Alexa 546). Bar 15 µm.
