# Supplementary material for: HbtR, a Heterofunctional Homolog of the Virulence Regulator TcpP, Facilitates the Transition between Symbiotic and Planktonic Lifestyles in Vibrio fischeri
Source: mBio. 2020 Sep 1;11(5):e01624-20. doi: 10.1128/mBio.01624-20 (PMC7468203; doi:10.1128/mBio.01624-20)
Supplement: TABLE S3 [file mBio.01624-20-st003.docx]

**Table S3. Bacterial strains and plasmids used in this work.**

**Strain Description Source**

ES114 *Vibrio fischeri*, wild-type (1)

BDB127 ES114 with empty pVSV105 This work

BDB128 ES114 with pVSV105::*hbtRC* This work

BDB171 ES114 with pVSV105::*litR* This work

BDB156 ES114 with pVSV102 (2)

BDB011 ES114 with pVSV208 (2)

CBNR46 ES114 *att*Tn*7*::*lacZp-gfp-erm* (3)

CBNR47 ES114 *att*Tn*7*::*lacZp-rfp-erm* (3)

BDB003 ES114 Δ*hbtRC* (VF_A0473‒A0474) (4)

BDB134 ES114 Δ*hbtRC* (VF_A0473‒A0474), newly derived This work

BDB039 Δ*hbtRC* (BDB003) with empty pVSV105 This work

BDB023 Δ*hbtRC* (BDB003) with pVSV105::*lacI*-*hbtRC* This work

BDB139 Δ*hbtRC* (BDB134) with empty pVSV105 This work

BDB140 Δ*hbtRC* (BDB134) with pVSV105::*hbtRC* This work

BDB154 Δ*hbtRC* (BDB134) with pVSV105::*tcpPH* This work

BDB157 Δ*hbtRC* (BDB134) with pVSV102 This work

BDB012 Δ*hbtRC* (BDB134) with pVSV208 This work

BDB078 Δ*hbtRC* (BDB003) *att*Tn*7*::*lacZp-gfp-erm* This work

BDB079 Δ*hbtRC* (BDB003) *att*Tn*7*::*lacZp-rfp-erm* This work

BDB172 Δ*hbtRC* Δ*toxRS* (VF_0790–0791) This work

BDB173 Δ*hbtRC* Δ*toxRS* with empty pVSV105 This work

BDB174 Δ*hbtRC* Δ*toxRS* with pVSV105::*hbtRC* This work

BDB182 ES114 Δ*litR* (VF_2177) This work

BDB183 Δ*litR* with empty pVSV105 This work

BDB184 Δ*litR* with pVSV105::*hbtRC* This work

BDB185 Δ*litR* with pVSV105::*litR* This work

BDB129 ES114 ΔVF0157–0180 This work

BDB186 ΔVF0157–0180 with empty pVSV105 This work

BDB187 ΔVF0157–0180 with pVSV105::*hbtRC* This work

BDB188 ΔVF0157–0180 with pVSV105::*litR* This work

BDB135 ES114 ΔVF_2042 This work

BDB141 ES114 ΔVF_1133 This work

BDB142 ES114 ΔVF_A0389 This work

BDB144 ES114 ΔVF_A0246 This work

BDB167 ES114 ΔVF_1133 ΔVF_A0246 This work

BDB163 ES114 ΔVF_1133 ΔVF_2042 ΔVF_A0246 ΔVF_A0389 This work

BDB130 ES114 Δ*aphB* (VF_1690) This work

JB24 ES114 Δ*crp* (VF_2280) (5)

CA1 ES114 Δ*rpoQ* (VF_A1015) (6)

BDB194 Δ*rpoQ* with empty pVSV105 This work

BDB195 Δ*rpoQ* with pVSV105::*hbtRC* This work

BDB176 Δ*rpoQ* with pVSV105::*litR* This work

0395-N1 *V. cholerae* Ogawa 395 Δ*ctxA* (7)

BDB190 0395-N1 Δ*tcpPH* (ΔVC0395_A0351-A0352) This work

BDB191 Δ*tcpPH* with empty pVSV105 This work

BDB192 Δ*tcpPH* with pVSV105::*hbtRC* This work

BDB193 Δ*tcpPH* with pVSV105::*tcpPH* This work

DH5α*pir E. coli* cloning strain; *recA1 endA1 hsdR17 supE44 thi-1* (8)

Δ*(lacZYA-argF)U169 [Φ80dlacZΔM15] gyrA96 relA1 λpir+*

WM3064 *E. coli* conjugation strain; *thrB1004 pro thi rpsL hsdS* (9)

*lacZΔM15 RP4-1360* Δ*(araBAD)567* Δ*dapA1341::[erm pir(wt)]*

**Plasmid Description Source**

pAKD601 IPTG-inducible expression vector (10)

pVSV105 Cloning vector; Cm^r^ (2)

pVSV105::*lacI-hbtRC lacI^q^*-*A1*/*O4*/*O3p* from pAKD601, This work

26 bp upstream of *lacI^q^*, 7 bp downstream of

*A1*/*O4*/*O3p*; VF_A0473‒A0474, 18 bp upstream,

48 bp downstream; Cm^r^

pVSV105::*hbtRC* VF_A0473‒A0474, 18 bp upstream, This work

48 bp downstream; Cm^r^

pVSV105::*litR* VF_2177, 21 bp upstream, 87 bp downstream; Cm^r^ This work

pVSV105::*tcpPH* VC0395_A0351-A0352, 20 bp upstream, This work

2 bp downstream; Cm^r^

pVSV102 *gfp*; Km^r^ (2)

pVSV208 *rfp*; Cm^r^ (2)

pEVS107 mini-Tn7 vector; Km^r^, Em^r^ (11)

pCBNR6 pEVS107::*lacZp-gfp*; Km^r^, Em^r^ (3)

pCBNR7 pEVS107::*lacZp-rfp*; Km^r^, Em^r^ (3)

pUX-BF13 *tnsABCDE* transposase vector (12)

pEVS104 Conjugation helper; Km^r^ (13)

pSMV3 Deletion vector; Km^r^, *sacB* (14)

pSMV3Δ*hbtRC* pSMV3 with VF_A0473‒A0474 flanking sequences This work

pSMV3Δ*litR* pSMV3 with VF_2177 flanking sequences This work

pSMV3ΔVF_0157-80 pSMV3 with VF_0157-0180 flanking sequences This work

pSMV3ΔVF_1133 pSMV3 with VF_1133 flanking sequences This work

pSMV3ΔVF_2042 pSMV3 with VF_2042 flanking sequences This work

pSMV3ΔVF_A0246 pSMV3 with VF_A0246 flanking sequences This work

pSMV3ΔVF_A0389 pSMV3 with VF_A0389 flanking sequences This work

pSMV3Δ*tcpPH* pSMV3 with VC0395_A0351-A0352 This work flanking sequences

1. Boettcher KJ, Ruby EG. 1990. Depressed light emission by symbiotic *Vibrio fischeri* of the sepiolid squid *Euprymna scolopes*. J Bacteriol 172:3701–3706.

2. Dunn AK, Millikan DS, Adin DM, Bose JL, Stabb EV. 2006. New *rfp*- and pES213-derived tools for analyzing symbiotic *Vibrio fischeri* reveal patterns of infection and *lux* expression *in situ*. Appl Environ Microbiol 72:802–810.

3. Bongrand C, Koch EJ, Moriano-Gutierrez S, Cordero OX, McFall-Ngai M, Polz MF, Ruby EG. 2016. A genomic comparison of 13 symbiotic *Vibrio fischeri* isolates from the perspective of their host source and colonization behavior. ISME J 10:2907–2917.

4. Thompson LR, Nikolakakis K, Pan S, Reed J, Knight R, Ruby EG. 2017. Transcriptional characterization of *Vibrio fischeri* during colonization of juvenile *Euprymna scolopes*. Environ Microbiol 19:1845–1856.

5. Bose JL, Kim U, Bartkowski W, Gunsalus RP, Overley AM, Lyell NL, Visick KL, Stabb EV. 2007. Bioluminescence in *Vibrio fischeri* is controlled by the redox-responsive regulator ArcA. Mol Microbiol 65:538–553.

6. Cao X, Studer SV, Wassarman K, Zhang Y, Ruby EG, Miyashiro T. 2012. The novel sigma factor-like regulator RpoQ controls luminescence, chitinase activity, and motility in *Vibrio fischeri*. mBio e00285-11.

7. Mekalanos JJ, Swartz DJ, Pearson GD, Harford N, Groyne F, de Wilde M. 1983. Cholera toxin genes: nucleotide sequence, deletion analysis and vaccine development. Nature 306:551–557.

8. Liss L. 1987. New M13 host: DH5 F’ competent cells. Focus 9:13.

9. Saltikov CW, Newman DK. 2003. Genetic identification of a respiratory arsenate reductase. Proc Natl Acad Sci U S A 100:10983–10988.

10. Dunn AK, Stabb EV. 2008. The twin arginine translocation system contributes to symbiotic colonization of *Euprymna scolopes* by *Vibrio fischeri*. FEMS Microbiol Lett 279:251–258.

11. McCann J, Stabb EV, Millikan DS, Ruby EG. 2003. Population dynamics of *Vibrio fischeri* during Infection of *Euprymna scolopes*. Appl Environ Microbiol 69:5928–5934.

12. Bao Y, Lies DP, Fu H, Roberts GP. 1991. An improved Tn7-based system for the single-copy insertion of cloned genes into chromosomes of gram-negative bacteria. Gene 109:167–168.

13. Stabb EV, Ruby EG. 2002. RP4-based plasmids for conjugation between *Escherichia coli* and members of the vibrionaceae, p. 413–426. *In* Methods in Enzymology. Academic Press.

14. Coursolle D, Gralnick JA. 2010. Modularity of the Mtr respiratory pathway of *Shewanella oneidensis* strain MR-1. Mol Microbiol 77:995–1008.
