# Supplementary material for: HbtR, a Heterofunctional Homolog of the Virulence Regulator TcpP, Facilitates the Transition between Symbiotic and Planktonic Lifestyles in Vibrio fischeri
Source: mBio. 2020 Sep 1;11(5):e01624-20. doi: 10.1128/mBio.01624-20 (PMC7468203; doi:10.1128/mBio.01624-20)
Supplement: TABLE S4 [file mBio.01624-20-st004.docx]

**Table S4. Primers and probes used in this work**

**Primer Sequence Restriction Site**

*lacI* and *hbtRC* insertion into pVSV105

lacIF2 CTAGGTCGACGCTAACTTACATTAATTGCGTTG *Sal*I

lacIR CTAGGAATTCCTGTGTGAAATTGTTATCCGC *Eco*RI

hbtRF CTAGGAATTCCTGTTAAGTCAGGATGATATGG *Eco*RI

hbtCR CATGGGTACCGTACCCTAAACACACTCTATAAATAAC *Kpn*I

*hbtRC* insertion into pVSV105

hbtRF2 CTAGTCTAGACTGTTAAGTCAGGATGATATGG *Xba*I

hbtCR CATGGGTACCGTACCCTAAACACACTCTATAAATAAC *Kpn*I

*litR* insertion into pVSV105

litRF GTACGTCGACGTTGGCAAGGATATAAATATAATGG *Sal*I

litRR GTACGGTACCTTCTGATTAACAACGCATTTG *Kpn*I

*tcpPH* insertion into pVSV105

VCtcpPF CATGTCTAGAGATTAAGAAAATGTAAAGTAATGG *Xba*I

VCtcpHR CATGGGTACCCCCTAAAAATCGCTTTGACAG *Kpn*I

Gene deletion

hbtRUSF GATCGGATCCGAGAGCATTAAGTAGTGTTAATC *Bam*HI

hbtRUSR GATCGGTACCCCATATCATCCTGACTTAACAG *KpnI*

hbtCDSF GATCGGTACCCAGCAGGAAGTGGTAGTCTC *Kpn*I

hbtCDSR GATCGAGCTCCAGGACTATTCGGGTTAAG *Sac*I

litRUSF GATCGGATCCGCATGATTAAGCGTTAAGATTAG *Bam*HI

litRUSR GATCGAATTCCCATTATATTTATATCCTTGCCAAC *Eco*RI

litRDSF GATCGAATTCCAAATTGTTTCTTAAATATGTTGTG *Eco*RI

litRDSR GATCGAGCTCGGTGGTATCATCGGTCTTG *Sac*I

VF1133USF GTACGGATCCGGTCACTTTGCACTCTCAC *Bam*HI

VF1133USR GTACGGTACCCTGTTCATGTTGTATCTCTCTTTC *Kpn*I

VF1133DSF GTACGGTACCGTAATTATCTTAGATAAGCAATGTC *Kpn*I

VF1133DSR GTACGAGCTCGCGTTTGGCATTGAGAATTG *Sac*I

VF2042USF GTACGAATTCGAAACCGTTTCAATCAAAACAG *Eco*RI

VF2042USR GTACGGTACCCATAGCTGCATACCTTAGTTATAAC *Kpn*I

VF2042DSF GTACGGTACCCTGTAATTAAGTTGATTTTTCACTTATG *Kpn*I

VF2042DSR GTACCTCGAGCCAGCTTTAATTGAGCCTTC *Xho*I

VFA0246USF GTACGGATCCCGCCTAATGCCTTCAATCG *Bam*HI

VFA0246USR GTACGGTACCCATAGATAACACCGCTGTAAG *Kpn*I

VFA0246DSF GTACGGTACCGTATAAAGGGTAATTCAATAGGCTG *Kpn*I

VFA0246DSR GTACGAGCTCCCAAGCCATCTCAATTTTACG *Sac*I

VFA0389USF GTACGGATCCGCCAGCCTTATCAGTTTTATTG *Bam*HI

VFA0389USR GTACGAATTCCTTCATAGAAACATCCTAAATCCG *Eco*RI

VFA0389DSF GTACGAATTCGTGTAACTGACGTATAACTATC *Eco*RI

VFA0389DSR GTACGAGCTCGCGTTACGACTATTGGTTTAC *Sac*I

VF0157USF GTACGGATCCGTCTAAAAATAAGAAAGCAGTGC *Bam*HI

VF0157USR GTACGGTACCGAAAAGGGCTTTGAGGGG *Kpn*I

VF0180DSF GTACGGTACCGATAAGCAAATAATGGCGTTTTG *Kpn*I

VF0180DSR GTACGAGCTCGAGTAACTTCGCTTAATGCC *Sac*I

VF1690USF GTACGGATCCCTGATCAATAAACAAGAGATAACG *Bam*HI

VF1690USR GTACGGTACCCTAATTTCATTATATTAAGATACTCTCC *Kpn*I

VF1690DSF GTACGGTACCGTAACCACATACCTGATTAATTTAC *Kpn*I

VF1690DSR GTACgagctcGTTTAGGTAAGCGTGAAATTG *Sac*I

toxRUSF GATCGGATCCGAACTCTTGGTATTCAGCATC *Bam*HI

toxRUSR GATCGGTACCCATCGGTTATTAACTCAGTAGTG *Kpn*I

toxSDSF GATCGGTACCCTATTTTCGCATTAACAATGATTTC *Kpn*I

toxSDSR GATCGAGCTCGAAATTTGGAAAAGATGACAGG *Sac*I

Expression analysis

VF1133expF CAACAAAGCGTCACTCAAAC

VF1133expR CCTCTAAGCTACGTGCAATC

VF2042expF CAGGTGAACAAGGTCGAG

VF2042expR CCTTCTTGGCAATCACGAC

VFA0246expF GGTGCAGAGTGAAGCTAATG

VFA0246expR TGAACCTTCAACGGTTAGTG

VFA0389expF GAACGTAGCATCAGGAGAAG

VFA0389expR GTGACTTAACGCTTTGGTTTC

flaAexpF GTCTGTAGGTGATGCACAAG

flaAexpR CGGCTGTTAGAAGCAGATAC

flaCexpF GGTGGTTCTCGTCTTCTTAATG

flaCexpR TCAGCATTTACAGCCCAATC

flaFexpF GGAAAGTAGGTGCAGAGAATAC

flaFexpR CGTCTCACCTGACATGAATAC

motA1expF CCTGCAATGGGTATGATTGG

motA1expR TGAGCAATAGGGAATGCAAC

flgMexpF CCAGAAACTCAAGCACCTG

flgMexpR CGTTCAGGATCAACTGTGTATG

flrAexpF TCGTGCAGATGTTCGTATTG

flrAexpR CTGCCTTCAGCTTCCATTC

flrBexpF ACCGCTCTGCAATCAAATC

flrBexpR TGACATCCCTTCCCTATCAC

VF0157expF CCAAATGTACGCTGGCTATC

VF0157expR CCATAATCACCGCCTTCTTG

VF0164expF GATTCTGCTGGTGTTGCG

VF0164expR GGCAAACGTCACTGGTTAG

VF0165expF GTACCAGTATGCACTTTGCC

VF0165expR CGGCATCCACAACCAATAC

hbtRexpF GCTGTTCCTGCAACAAGTAG

hbtRexpR GAGACTACCACTTCCTGCTG

litRexpF GGAGTACCTCTACTCGTG

litRexpR CAGGGGTATGATCGTTAC

toxTexpF AAAGAACTAGAGTCTCGAGGAG

toxTexpR ACTTGGTGCTACATTCATGG

VCpolAqF AAGAGCTGGCTTTGGATTAC

VCpolAqR CCGTCACTGCTTGATGATAG

*In situ* HCR probes targeting *hbtR*

ATGGTTTTTAAACTCAATGAAATCTATTGGGATCCAGCAACAAAAAAATTGT

GATACGCTAGAAGATGCAGTTAATGGTAATAACGAATATGGTTCAACAACGC

TTAGTTTCTGCAATTCTGACTTTGTTAATAAAAGAACATCCTTCTATTTGTA

AATGAACATATTAAAGATGCTCTTTGGGGGACTCAGTGGATATCAAATGAAA

ATTCCTCAGTTAATTAAAAGAACAAGAGTATCAATAAAAGATACGAATAGAC

GTTATCGAAAATGTAAAAGGCAATGGTTATAAAATAAACAATGTAGAAGAGA

AGAATAAATTCAAATAGCATACAATGCACAGAGAAAAAAACGTCACATTCTA

TCAGCATTAATCTTTTGTATAGAAAAACATGTGTTTTACGATCTAATTCCTT

GAAGAGATAAAAAAAGTCAAAGATTTTGATTTAATTAAAATCTCTGAAGATA

TTTTTAATTAAGACTAAAGACCAAGAGTGTGAATTGAACCTTAAAAATAAAA

*In situ* HCR probes targeting *litR*

ATGGATACAATCCAAAAAAGGCCTAGAACAAGGCTATCTCCAGAAAAGCGCA

GAACAATTACTTGATATCGCAATTGAAGTGTTTTCACAACGTGGCATTGGTC

GGTGGTCATGCTGATATTGCAGAAATCGCTCAGGTCTCTGTCGCTACCGTGT

AATTACTTCCCAACAAGAGAAGATCTTGTTGATGATGTATTAAATAAAGTCG

AACGAATTTCACCAATTCATCAATAACTCAATTTCTCTTGATCTTGATGTTC

TCTAACCTTAATACTCTTCTTCTGAATATCATTGACAGTGTTCAAACTGGTA

AAGTGGATCAAAGTATGGTTTGAATGGAGTACCTCTACTCGTGATGAAGTAT

CCTCTTTTCTTAAGTACTCACTCAAATACAAATCAAGTTATCAAAACTATGT

GAAGAAGGTATTGAACGTAACGAAGTATGTAACGATCATACCCCTGAAAACC

ACAAAAATGCTTCATGGTATTTGTTACTCTGTTTTCATTCAGGCTAACCGTA

AGTTCATCTGAAGAAATGGAAGAAACGGCAAATTGTTTCTTAAATATGTTGT
